# Medical Electricity: Embracing Electro-Physiology and Electricity as a Therapeutic, with Special Reference to Practical Medicine; Showing the Most Approved Apparatus, Methods, and Rules for the Medical Uses of Electricity in the Treatment of Nervous Diseases

**Published:** 1866-09

**Authors:** 


MEDICAL ELECTRICITY: EMBRACING ELECTRO-PHYSIOLOGY AND ELECTRICITY AS A THERAPEUTIC, WITH SPECIAL REFERENCE TO PRACTICAL MEDICINE; SHOWING THE MOST APPROVED APPARATUS, METHODS, AND RULES FOR THE MEDICAL USES OF ELECTRICITY IN THE TREATMENT OF NERVOUS DISEASES. By ALFRED C. GARRATT, M.D., Fellow of the Mass. Med. Society; Member of the Amer. Med. Association. Third Edition, revised and illustrated. PHILADELPHIA: J. B. LIPPINCOTT & Co. 1866.

This is a ponderous volume of 1103 pages, published in good style, on good type and paper, and illustrated by 138 cuts.

The work is divided into twelve chapters, embracing the following subjects:—Natural Electricity; Early History of the Medical Uses of Electricity; Electro-Physiology; Electrical Instruments and Apparatus for Medical Purposes; Methods for the Employment of Electricity; Hyperæsthesia; Anæsthesia; Spastic Diseases; Midwifery; Nervous Affections of the Thorax and Abdomen; Feigned Nervous Affections—Sea Sickness; Electricity in Surgery. These headings, with the copious title-page, are sufficient to indicate the extent and great importance of this volume. The subjects of which it treats are among the most interesting and important, both in a physiological and therapeutic view, that can engage the attention of the student and practitioner. How far the author of the present treatise has succeeded in presenting the facts, at present known to the scientific portion of the profession, relating to each topic discussed, we cannot say; for our time has been too closely occupied with the practical duties of our profession to enable us to give the work a careful perusal. Every student and practitioner, however, will be abundantly repaid for the purchase and careful study of the work.

